# Recent self-harm and psychological measures in the emergency department

**DOI:** 10.7717/peerj.667

**Published:** 2014-11-11

**Authors:** Jason R. Randall, Brian H. Rowe, Kathryn A. Dong, Ian Colman

**Affiliations:** 1 School of Public Health, University of Alberta, Edmonton, Canada; 2 Department of Community Health Sciences, College of Medicine, Faculty of Health Sciences, University of Manitoba, Winnipeg, Canada; 3 Department of Emergency Medicine, Faculty of Medicine and Dentistry, University of Alberta, Edmonton, Canada; 4 Department of Epidemiology and Community Medicine, University of Ottawa, Ottawa, Canada

**Keywords:** Risk assessment, Emergency medicine, Self-injurious behaviors, Psychiatry

## Abstract

The assessment of self-harm risk is a common, difficult, and perplexing task for many physicians, especially those working in emergency departments (ED). Attempts have been made to determine objective methods for assessing patients with suicidal ideation or self-harm though there is still a lack of knowledge about objective assessments of these patients. A study was conducted where 181 suicidal patients were enrolled in two EDs within the city of Edmonton, Canada. Initial interviews were conducted in the ED which collected basic demographics and medical history as well as psychometric measures including the Beck Hopelessness Scale, Barratt Impulsiveness Scale, Brief Symptom Inventory, Drug Abuse Screening Test 10, and CAGE questionnaire. The results of these measures were compared between those who presented to the ED with self-harm and those who presented only with ideation. Those with recent self-harm scored lower on many of the scales and subscales of distress and impulsivity measured compared to those with no recent self-harm. Possible explanations for this difference include differences in psychological traits between the two groups and possible cathartic effects of self-harm. The lower scores obtained by those that present with self-harm may complicate attempts to use psychometric tools to determine future self-harm risk.

## Background

The assessment of suicidal and self-harming patients is a common occurrence in primary care (Gaynes et al., 2004) particularly in the emergency department (ED; Doshi et al., 2005). There has been research on psychometric tools and their relationship with self-harm and suicide (Hockberger & Rothstein, 1988; Feinstein & Plutchik, 1990; Bullard, 1993; Cremniter et al., 2001; Cooper et al., 2006; McAuliffe et al., 2008; Nock et al., 2010; Bolton, Spiwak & Sareen, 2012). There are limited data available on psychometric measures related to self-harm presentations in acute settings like the ED (Randall, Colman & Rowe, 2011). In order to maximize the effectiveness of psychometric assessment tools clinicians need to understand how they relate to patient characteristics, such as their history of self-harm, at presentation. These characteristics may affect the scores on these tools and confound their ability to assess the risk of future self-harm.

There has been considerable debate over the proper terminology to use when describing suicide and related behaviours. This paper uses ‘self-harm’ to mean any form of intentional self-injury regardless of whether there was suicidal intent or not. This usage is derived from a WHO study on parasuicide (Platt et al., 1992), and is more commonly used in European research. When discussing other literature, the terms non-suicidal self-injury (NSSI), suicide attempts, and suicide will be used where appropriate.

If self-harm is a response to distress, possibly as a means of self-regulation or as a “cry of pain” as theorized by various researchers (O’Connor, 2003; Skegg, 2005; Klonsky, 2007; Klonsky, 2009; Nock, 2010; Rasmussen et al., 2010), then it should occur at a high point in psychological distress. Therefore it is expected that measures of distress (e.g., presence of acute psychological symptoms) would be higher in those who present with recent self-harm compared to others who may be contemplating self-harm. Others have suggested that self-harming may have a cathartic effect that results in reduced levels of distress, though opinion is mixed on this matter (Van Praag & Plutchik, 1985; Bronisch, 1992; Walker, Joiner & Rudd, 2001; Jallade, Sarfati & Hardy-Baylé, 2005; Franklin et al., 2010). If such an effect exists, it’s not clear how soon it may appear in the aftermath of the self-harming event, and what effect they may have on ED risk assessment of future suicidal behaviour.

This study examines the characteristics of a group of patients at risk for future self-harm that presented to two EDs in Edmonton, Canada. We hypothesized that psychometric measures would have higher scores among those who were being treated for a recent self-harm event, versus those presenting with ideation only. A lack of a positive correlation could indicate that the additional risk in the patients presenting with self-harm is not due to higher distress, but rather by some other characteristic not measured by these questionnaires. This would indicate that these questionnaires are of limited value in isolation and that a more complicated method of assessing self-harm combining clinical and psychometric measures is required. The association between previous history of self-harm (not recent) and the questionnaires was also assessed.

## Methods

### Sample

Patient enrolment occurred within the EDs of the Royal Alexandra Hospital and the University of Alberta Hospital in Edmonton, Canada. Study enrolment began in August 2009 and was completed in May 2010. These sites are the two largest EDs and teaching hospitals in the Edmonton region and are staffed by full-time ED physicians. Both EDs teach rotating interns and residents and have fulltime day ED psychiatric staff and inpatient mental health services.

Patients presenting to these EDs with self-harm or ideation were enrolled while they were being assessed in the ED. Only permanent residents of Alberta were enrolled (to increase follow up rates for the prospective portion of the study) and enrolment was limited to the adult population (age >17). We excluded patients who were violent, did not have the capacity to provide informed consent to the study (e.g., dementia, cognitive impairment), were suffering from an acute medical condition that would prevent them from participating (e.g., intentional drug overdose, coma, etc.), and/or those who were unable to understand or communicate in English. Eligibility for the study was determined by the most responsible physician; normally this was an ED physician, a psychiatrist or a psychiatry resident.

### Assessments

Patients who agreed to study enrolment were administered a verbal interview questionnaire that collected relevant demographic (e.g., age, gender, education level, living situation) and medical (e.g., history of psychiatric disorders and self-harm) information. The patients then completed several written questionnaires.

The Brief Symptom Inventory (BSI; Derogatis & Melisaratos, 1983) is a 53-item questionnaire that assesses a variety of psychiatric symptoms present in the past week using a five point rating scale. Subscales of this questionnaire measure anxiety, depression, hostility, interpersonal sensitivity, obsessive compulsivity, paranoid ideation, phobic anxiety, psychoticism and somatisation. The Barratt Impulsiveness Scale (BI; Patton, Stanford & Barratt, 1995) is a 30-item questionnaire designed to measure a person’s level of impulsiveness using a four point rating scale. The questionnaire provides several subscales that attempt to measure specific aspects of impulsiveness including attentional, attentional impulsiveness, cognitive complexity, cognitive instability, motor, motor impulsiveness, non-planning impulsiveness, perseverance and self-control. The Beck Hopelessness Scale (BHS; Beck et al., 1974) is a 20-item true or false questionnaire that attempts to measure the level of hopelessness that a patient has about their future. The CAGE (Ewing, 1984) questionnaire is a 4-question alcohol abuse screen with a cut off score of 3 or greater while the Drug Abuse Screening Test-10 (DAST; Skinner, 1982) is a 10-item questionnaire that measures the use of drugs. A Manchester Self-harm rule (Cooper et al., 2006) risk assessment was calculated by the researcher at the time of the interview based on information gathered from the patient and medical staff. The Manchester Self-harm rule contains four screening questions where a positive response to any question entails a positive result.

### Analysis

Failure to answer one or more of the questionnaire items was adjusted for during analysis. The method of scoring for the BSI adjusted for missing answers by averaging the scores of the responses (Derogatis, 2006). The BHS and BI do not have explicitly stated methods to deal with missing values; however, the same method used for the BSI was applied to questionnaires that had 90% or more of the questions answered. For the BI this method was not possible due to the scoring method of the measurement so it was decided that mean replacement would be used for missing questions. As with the BHS questionnaire, those BI questionnaires that were less than 90% completed were counted as missing. The pattern of missing values was analyzed for the questionnaires and there were no indication that the missing values would affect the results. Continuous variables were assessed for suitability for analysis as continuous variables and data which were not suitable for analysis as a continuous variable were grouped.

Patients were grouped by two dichotomous variables: presentation with recent self-harm, and history of self-harm. For the presentation variable, it was determined whether the patient presented with recent self-harm (self-harm occurring shortly before ED presentation and requiring treatment and/or instigating their presentation), or the patient presented without recent self-harm. Secondly, for the history of self-harm variable, it was determined whether the patient had a history of prior self-harm, not including self-harm treated during current presentation, or no prior history. First, ANOVA or regression analysis was performed using a categorical variable with all 4 possible subgroup combinations of the two variables of interest (history of self-harm, presentation with recent self-harm). ANOVA was used to analyze the questionnaire data. Logistic regression analysis was used for binary variables (e.g., history of mood disorder) and multinomial logistic regression was used for categorical variables (e.g., age category). If a significant *p*-value was obtained for the overall model, ANOVA or logistic regression was used to determine which of the two variables were individually significant.

### Post-hoc analysis

A measure of distress was created using the questionnaire results that were significantly different between the self-harm and ideation groups. This measure was derived by combining the results of the significant questionnaire results with each scale having equal weight. This measure of distress was plotted against the length of time that the patients had been in the ED before they were assessed by the research team. A lowess smoother (a scatterplot with a line used to estimate the data trend) was used to determine the trend for the self-harm and ideation groups. Multiple imputation was used to estimate the missing values for the distress variable and then regression analysis was performed to assess whether demographic or medical history variables collected were influencing the relationship between a patients distress score and their presentation status (i.e., self-harm versus ideation only). The demographic variables entered into the regression analysis were education level, age, marital states, living arrangements (e.g., living alone, living with family etc.), and gender. The medical history variables were lifetime history of self-harm as well as a history of mood disorders, personality disorders, psychotic disorders, bipolar disorder and another disorder variable containing any previous diagnosis that did not fit into one of the previously mentioned diagnostic groups. The purpose of these analyses was to assess possible explanations of the results of the regression analysis.

### Ethics

This study received approval from the Human Research Ethics Board of the University of Alberta (HERO #: Pro00005188). Informed written consent was obtained from each participant.

## Results

### Sample

A total of 181 patients agreed to participate in the study, while 89 patients refused to participate. Complete information was obtained for a total of 157 patients. Participants in the study did not differ from those who refused based on gender, age, occurrence of self-harm at presentation, psychiatry consultation, and disposition (population characteristics described in Table 1). There was no relationship between failure to complete the questionnaires and the demographic characteristics measured.

**Table 1:** Characteristics of sample versus refusal group.

		Study sample		Refused	
Variable	Category	*N*	%	*N*	%
**Gender**	Male	93	51.4	49	55.1
	Female	87	48.1	40	44.9
	Transgendered	1	0.6	–	–
**Age**	18–29	65	35.9	35	39.3
	30–45	64	35.4	30	33.7
	45 +	52	28.7	24	27.0
Method of self-harm*	OD/Poisoning	55	30.6	26	33.8
	Hanging/Suffocation	5	2.8	1	2.7
	Laceration/Puncture	21	11.7	8	10.4
	All other	6	3.3	4	2.9
	Any self-harm	82	45.6	37	44.5
**Psychiatry consult**	Yes	158	87.3	71	79.8
	No	23	12.7	18	20.2
**Admitted**	Admitted	90	49.7	35	39.3
	Discharged	91	50.3	54	60.7

### Questionnaire results

Those who presented with recent self-harm were compared to those without recent self-harm to determine if any significant differences existed between those two groups. Those with recent self-harm scored significantly lower on several questionnaire scores compared to those without recent self-harm (Table 2). For the BSI, the Global Severity Index (GSI; *p* = 0.020), Positive Symptom Distress Index (PSDI; *p* = 0.023), Positive Symptom Total (PST) (*p* = 0.026), Anxiety (*p* = 0.020), Depression (*p* = 0.014), Hostility (*p* = 0.003), Interpersonal Sensitivity (*p* = 0.036), Obsessive Compulsive (*p* = 0.005) and Phobic Anxiety (*p* = 0.032) were significantly different between these two groups (self-harm patients endorsed less symptoms than those without self-harm). The BI found the Attentional Impulsiveness (*p* = 0.026), Cognitive Instability (*p* = 0.015), were significantly lower in those presenting with recent self-harm. The two groups also differed in their Beck Hopelessness Scale scores (*p* = 0.012). The DAST-10 scale and CAGE questionnaire were not significant.

**Table 2:** Questionnaire score comparison of means for those presenting with self-harm.

	Sample mean	Presented with self-harm			
		**Yes** ***N*** = **82**		**No** ***N*** = **98**	
**Questionnaire/scale**	**Mean**	**Mean**	**SD**	**Mean**	**SD**
Beck hopelessness scale	13.2	^*^ **12.1**	6.1	^*^ **14.3**	4.9
DAST-10	2.7	2.5	3.1	2.7	3.1
CAGE	1.5	1.4	1.6	1.4	1.6
SAD PERSONS	4.4	4.5	1.7	4.2	1.6
Manchester self-harm rule	N/A	0.85	0.4	0.88	0.33
**Brief Symptom Inventory**	**Mean**	**Mean**	**SD**	**Mean**	**SD**
Global severity index	2.4	^*^ **2.2**	1.0	^*^ **2.5**	0.7
Positive symptom distress index	2.9	^*^ **2.8**	0.7	^*^ **3.0**	0.6
Positive symptom total	37.0	^*^ **34.1**	17.5	^*^ **39.4**	15.0
Anxiety	2.5	^*^ **2.3**	1.2	^*^ **2.6**	1.0
Depression	3.1	^*^ **2.9**	1.1	^*^ **3.3**	0.7
Hostility	2.1	^**^ **1.8**	1.2	^**^ **2.3**	1.1
interpersonal sensitivity	2.6	^*^ **2.4**	1.2	^*^ **2.7**	0.3
Obsessive compulsive	2.6	^*^ **2.2**	1.2	^*^ **2.8**	0.9
Paranoid ideation	2.0	1.9	1.2	2.1	1.0
Phobic anxiety	1.8	^*^ **1.6**	1.3	^*^ **2.0**	1.2
Psychoticism	2.3	2.1	1.1	2.4	0.9
Somatization	1.9	1.7	1.2	2.0	1.0
**Barratt impulsiveness score**	**Mean**	**Mean**	**SD**	**Mean**	**SD**
Total score	76.4	74.4	14.9	78.0	12.5
Attention	12.4	11.9	3.2	12.7	3.4
Attentional impulsiveness	19.9	^*^ **19.0**	4.8	^*^ **20.7**	4.5
Cognitive complexity	13.6	13.3	2.7	12.9	2.7
Cognitive instability	7.6	^**^ **7.1**	2.4	^**^ **8.0**	2.0
Motor	17.6	17.8	4.5	17.5	4.4
Motor impulsiveness	27.2	27.1	6.2	27.5	5.8
Non-planning impulsiveness	29.3	28.4	6.1	29.8	5.7
Perseverance	9.6	9.2	2.5	10.0	2.3
Self-control	15.6	15.1	4.1	15.9	3.8

**Notes.**

* *F* statistic *p*-value <0.05.

** *F* statistic *p*-value <0.01.

*** *F* statistic *p*-value <0.001.

The questionnaire scores of those with a history of previous self-harm were also compared to those without a history in Table 3. The mean differences were examined and many of the scales were found to have significant differences. For the BSI the Global Severity Index (GSI; *p* < 0.001), Positive Symptom Total (PST; *p* = 0.003), Anxiety (*p* = 0.006), Depression (*p* < 0.001), Hostility (*p* < 0.001), Interpersonal Sensitivity (*p* = 0.006), Obsessive Compulsive (*p* = 0.037), Paranoid Ideation (*p* = 0.003), Phobic Anxiety (*p* < 0.001), Psychoticism (*p* < 0.001) and Somatization (*p* = 0.024) were significantly higher in the group with a history of self-harm. Analysis of the BI found that only the cognitive complexity subscale failed to achieve statistical significance. All of the remaining subscales were significantly higher for the group with a history of self-harm. Of the remaining three questionnaires, the DAST-10, Beck Hopelessness Scale and CAGE questionnaire, no significant differences were identified.

**Table 3:** Questionnaire score comparison of means for those with previous history of self-harm.

	**Sample mean**	History of self-harm			
		**Yes** ***N*** = **130**		**No** ***N*** = **49**	
**Questionnaire/scale**	**Mean**	**Mean**	**SD**	**Mean**	**SD**
Beck hopelessness scale	13.2	13.8	5.3	12.2	6.2
DAST-10	2.7	3.0	3.1	2.0	2.9
CAGE	1.5	1.6	1.6	1.1	1.6
SAD PERSONS	4.4	^***^ **4.8**	1.5	^***^ **3.1**	1.3
Manchester self-harm rule	N/A	N/A	N/A	N/A	N/A
**Brief symptom inventory**	**Mean**	**Mean**	**SD**	**Mean**	**SD**
Global severity index	2.4	^***^ **2.5**	0.8	^***^ **2.0**	1.0
Positive symptom distress index	2.9	2.9	0.6	2.8	0.7
Positive symptom total	37.0	^**^ **38.6**	16.1	^**^ **33.4**	15.7
Anxiety	2.5	^**^ **2.6**	1.1	^**^ **2.1**	1.2
Depression	3.1	^***^ **3.3**	0.8	^***^ **2.7**	1.2
Hostility	2.1	^***^ **2.3**	1.1	^***^ **1.6**	1.2
Interpersonal sensitivity	2.6	^**^ **2.7**	1.0	^**^ **2.2**	1.2
Obsessive compulsive	2.6	^*^ **2.7**	1.0	^*^ **2.3**	1.2
Paranoid ideation	2.0	^**^ **2.3**	1.0	^**^ **1.6**	1.1
Phobic anxiety	1.8	^***^ **2.0**	1.2	^***^ **1.3**	1.3
Psychoticism	2.3	^***^ **2.5**	0.9	^***^ **1.9**	1.1
Somatization	1.9	^*^ **2.0**	1.1	^*^ **1.6**	1.1
**Barratt impulsiveness score**	**Mean**	**Mean**	**SD**	**Mean**	**SD**
Total score	76.4	^***^ **78.6**	13.4	^***^ **71.5**	12.7
Attention	12.4	^**^ **12.8**	3.3	^**^ **11.3**	3.2
Attentional impulsiveness	19.9	^**^ **20.6**	4.6	^**^ **18.3**	4.4
Cognitive complexity	13.6	13.9	2.7	12.9	2.5
Cognitive instability	7.6	^**^ **7.8**	2.2	^**^ **6.9**	2.1
Motor	17.6	^*^ **18.1**	4.5	^*^ **16.7**	4.3
Motor impulsiveness	27.2	^*^ **28.0**	5.9	^*^ **25.9**	5.9
Non-planning impulsiveness	29.3	^**^ **30.0**	5.7	^**^ **27.3**	6.0
Perseverance	9.6	^*^ **9.9**	2.4	^*^ **9.1**	2.4
Self-control	15.6	^**^ **16.1**	3.8	^**^ **14.4**	4.0

**Notes.**

* *F* statistic *p*-value <0.05.

** *F* statistic *p*-value <0.01.

*** *F* statistic *p*-value <0.001.

### Post-hoc analysis

**Figure 1:**
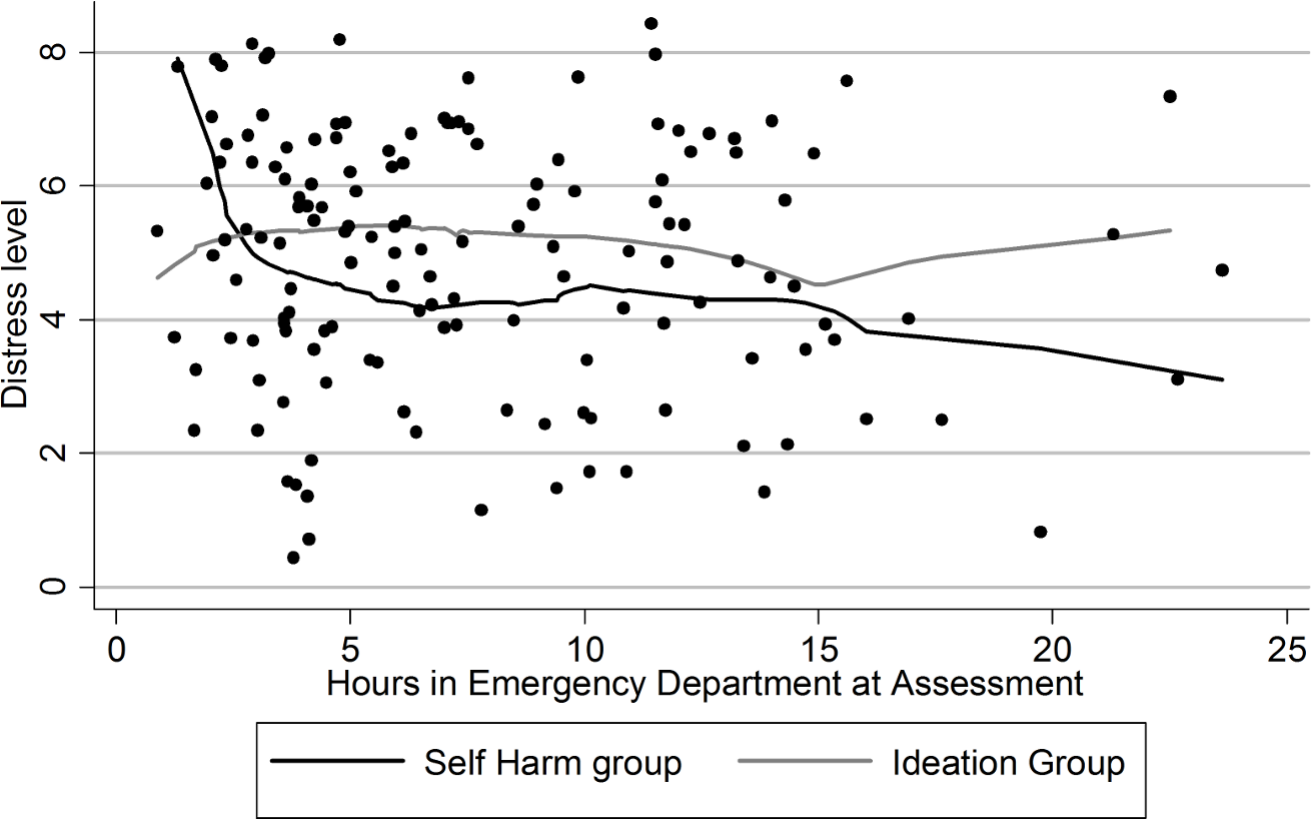
Distress level of patients. Self-harm group versus ideation only group.

The post-hoc analysis revealed a decrease in distress over the first hours in the ED for those that had engaged in self-harm (Fig. 1). However this trend was not significant. There was no apparent trend in the level of distress for patients that presented with ideation only. The regression analysis found no evidence that the result is due to confounding from extraneous variables. Entering the other potentially relevant variables not only failed to decrease the strength of the noted association between the questionnaires and recent self-harm but instead increased the strength of the relationship slightly. A similar pattern occurred when the individual scales were regressed instead of the aggregate measure of distress.

## Discussion

In this group of 181 patients at risk for future self-harm, high scores on several psychometric questionnaires were associated with recent self-harm. Interestingly, those who presented with recent self-harm scored considerably lower than those presenting with no recent self-harm. There are several potential explanations for this result. One explanation is that engaging in self-harm can have an effect similar to catharsis and afterwards these patients may feel less psychologically distressed than similar patients who did not engage in self-harm recently. Another possibility is that this is due to the trait differences between the patient groups in the study. Both explanations suggest that patient history should be taken into account when interpreting the scores of these questionnaires.

While it is possible that there is some form of cathartic effect at work for those who engaged in self-harm, overall the evidence for cathartic affects in self-harm is mixed (Van Praag & Plutchik, 1985; Bronisch, 1992; Walker, Joiner & Rudd, 2001; Jallade, Sarfati & Hardy-Baylé, 2005; Franklin et al., 2010). Evidence against this particular cause is provided in previous research by Walker, Joiner & Rudd (2001) that showed that the presence of a cathartic effect is not immediate after the self-harm but takes the form of an improvement in the time period after the self-harm. The authors suggested that the effect is not genuine catharsis but rather that it is a gradual decrease in suicidality due to increased interpersonal support after a self-harm event.

Other studies using matched samples of admitted attempters and admitted non-attempters provided mixed results (Van Praag & Plutchik, 1985; Bronisch, 1992). One study found a significant decrease in depression rating among those admitted after a suicide attempt but no decrease in those admitted for depression, despite initially similar scores (Van Praag & Plutchik, 1985). However an attempt to replicate this study failed to find the same result (Bronisch, 1992). Another study found a decrease in distress 8 days after hospitalization for a deliberate overdose (Sarfati, Bouchaud & Hardy-Baylé, 2003). However this study had no control group to rule out other explanations (e.g., regression to the mean or response to treatment; Sarfati, Bouchaud & Hardy-Baylé, 2003). An attempt to replicate the results with a control group did find a significant effect consistent with catharsis (Jallade, Sarfati & Hardy-Baylé, 2005). However this study utilized people involved in accidental traumas as the control group despite these individuals having consistently better scores on the distress level at baseline.

More support for a possible cathartic effect comes from a recent study that detected an immediate effect consistent with a cathartic effect (Franklin et al., 2010). This study used a proxy (cold water immersion) for NSSI and found that this proxy decreased the level of arousal and improved cognitive processing in both subjects with a history of NSSI and in control groups that were also exposed to the proxy. Measures of psychological distress did not detect a difference in the patients due to the NSSI proxy; however, it is possible that this is a side effect of the proximity of the measurement and the NSSI proxy. In our study there was a longer gap in time between the occurrence of the self-harm and the measurements of distress than in the study by Franklin et al. (2010). This may indicate that, while self-harming serves to rapidly reduce arousal states, it may take some time for the reduction in arousal to translate to a reduction in the patients subjectively experienced state of psychological distress. If this is the case then a delayed decrease in distress levels over time should be detectable in those patients who had engaged in self-harm but not in those with ideation only. Those patients that were assessed closer to the self-harm event should have higher levels of distress than the ideation group. This level of distress should decrease until the self-harm group begins to have significantly lower levels of distress compared to the ideation group. A post-hoc graph obtained shows the pattern that would be expected based on this explanation (Fig. 1). Though, due to the small sample size and post hoc nature of the analysis, it cannot be known for certain whether this trend is real as there was insufficient power to determine the significance of the trend. It is possible that other patient factors or factors in the ED environment may be causing the decrease in distress in those with recent self-harm though none of the possible confounders measured by this study could explain the effect.

Another explanation is that the difference detected was present before the patients had engaged in self-harm. One explanation within this framework is that these results are indicative of a difference between traits in this population and that the two groups (recent self-harm and ideation only) would have shown a similar disparity in the psychometric results before the self-harm event as well. It is possible that the higher scores obtained by the ideation group would be largely the result of a persistent trait of psychological distress or neuroticism. It is also possible that a portion of the patients with self-harm had engaged in this behaviour due to factors other than mental illness and psychological distress. The root causes of their self-harm might not be detected by any of the measures used in this study and may be confounding the results. Another study found that measures of trait neuroticism and openness were significantly different between patients who attempted suicide and patients who had completed suicide (Useda et al., 2007). They found that neuroticism was negatively correlated with completed suicide. However an additional study found that high levels of neuroticism resulted in a higher rate of suicidal ideation and suicide attempts (Fergusson, Woodward & Horwood, 2012). It is possible that these or similar traits may have been a factor but it is unclear with the present state of research in this area. This study attempted to adjust for the effects of patient traits on the relationship between the questionnaires and recent self-harm. Many possible confounders were entered in the regression analysis but they did not have any effect on the significance of the relationship between questionnaire scores and recent self-harm.

This study illustrates that it is necessary to take into account the presentation characteristics of individuals in the ED and to use that information, in addition to the scores from psychological assessments, in order to improve patient assessment. Recent self-harm and high scores on many of the questionnaires were later found to be correlated with future incidents of self-harm (Randall, Rowe & Colman, 2012). Without adjusting for the occurrence of recent self-harm it is likely that the effectiveness of psychometric measures could be underestimated in this population. This also indicates that more in-depth understanding of the confounding factors (in this instance the occurrence of recent self-harm) influencing patients’ psychological state may be necessary to form a comprehensive method of objective self-harm risk assessment. Further research should attempt to determine which of the proposed explanations is involved in producing the pattern of results found in the literature. Further research should try to determine methods of assessment that are applicable to all patients that present at risk for self-harm. As this study indicates different scoring mechanisms may be needed for different patient types. The easiest way to adjust scoring is by using predictive models including all relevant predictive and confounding variables that are available for assessment.

### Limitations

As this is a cross sectional study, caution should be taken with attempts to determine any causal patterns from the data provided as directionality of any established relationship cannot be ascertained with a high degree of certainty. Due to the large number of comparisons there is a risk of Type I error for the differences detected in this paper. For differences such as those detected in the questionnaire mean scores, however, the chance that these scores are spurious is reduced by the high agreement between multiple questionnaires and scales.

This study also represents a convenience sample of patients. Attempts were made to determine if these patients were different than those that were not included in the study and these differences appear to be minimal; however, it is still possible that the enrolled patients may differ from those that refused to join the study or missed patients in significant ways. This study was also not capable of enrolling patients who presented with high risk self-harm attempts as these patients were often admitted to the ICU or were otherwise ineligible for study participation. It is therefore uncertain if those who present with high risk self-harm incidents were different from those who were described in this study. Another noteworthy aspect of this study was that our measurement of self-harm did not distinguish between suicidal and non-suicidal self-injury. Additionally, there were some missing values in the questionnaires results but analysis of the results did not show any apparent bias that would result from them.

## Conclusion

The questionnaire results seem to reinforce the idea that there are differences among these patients based on the patients’ history of self-harming behaviours. However, the questionnaire results from this study were contrary to expectations that those presenting with recent self-harm would be more psychologically distressed than those presenting with ideation alone. This finding may be due to a type of “cathartic effect” experienced by those with recent self-harm. It also could be due to an unrelated effect of treatment (e.g., reduced distress due to increased social support in the ED or by family). Another possibility is that there are inherent differences between the type of patients that present with self-harm and those that present with ideation only. Further exploration of this discrepancy and its causes may assist in the assessment of self-harm risk for patients with recent self-harm. Future research on assessment methods in acute settings should focus on assessing all patients at risk rather than solely those who present with recent self-harm. A thorough clinical history should be combined with psychometric measures to ensure all confounders and relevant covariates can be identified. Combining psychometric and clinical factors into a weight scoring system should produce superior assessments and maximize the usefulness of clinically relevant psychometric tools.
